# An EST database from saffron stigmas

**DOI:** 10.1186/1471-2229-7-53

**Published:** 2007-10-09

**Authors:** Nunzio D'Agostino, Daniele Pizzichini, Maria Luisa Chiusano, Giovanni Giuliano

**Affiliations:** 1 Department of Soil, Plant, Environmental and Animal Production Sciences, University of Naples Federico II, via Università 100 - 80055 Portici (NA), Italy; 2 ENEA, Casaccia Research Center, PO Box 2400, Roma 00100AD, Italy

## Abstract

**Background:**

Saffron (*Crocus sativus *L., Iridaceae) flowers have been used as a spice and medicinal plant ever since the Greek-Minoan civilization. The edible part – the stigmas – are commonly considered the most expensive spice in the world and are the site of a peculiar secondary metabolism, responsible for the characteristic color and flavor of saffron.

**Results:**

We produced 6,603 high quality Expressed Sequence Tags (ESTs) from a saffron stigma cDNA library. This collection is accessible and searchable through the *Saffron Genes *database http://www.saffrongenes.org. The ESTs have been grouped into 1,893 Clusters, each corresponding to a different expressed gene, and annotated. The complete set of raw EST sequences, as well as of their electopherograms, are maintained in the database, allowing users to investigate sequence qualities and EST structural features (vector contamination, repeat regions). The saffron stigma transcriptome contains a series of interesting sequences (putative sex determination genes, lipid and carotenoid metabolism enzymes, transcription factors).

**Conclusion:**

The *Saffron Genes *database represents the first reference collection for the genomics of Iridaceae, for the molecular biology of stigma biogenesis, as well as for the metabolic pathways underlying saffron secondary metabolism.

## Background

Saffron (*Crocus sativus L*.) is a triploid, sterile plant, probably derived from the wild species *Crocus cartwrightianus*. It has been propagated and used as a spice and medicinal plant in the Mediterranean area for thousands of years [1]. The domestication of saffron probably occurred in the Greek-Minoan civilization between 3,000 and 1,600 B.C. A fresco depicting saffron gatherers, dating back to 1,600 B.C. has been unearthed on the island of Santorini, Greece.

**Figure 1:**
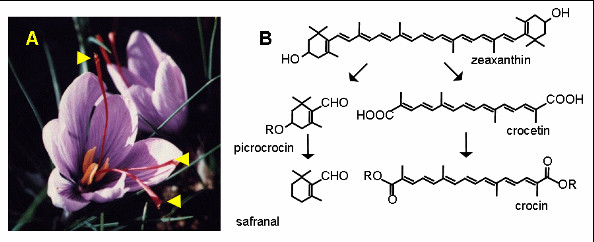
**The saffron spice**. A. Crocus flowers. Arrowheads point to the stigmas, which, harvested and desiccated, constitute the saffron spice. B. Biosynthetic pathway of the main saffron color (crocin) and flavors (picrocrocin and safranal) (from [2], modified).

Saffron is commonly considered the most expensive spice on earth. Nowadays, the main producing countries are Iran, Greece, Spain, Italy, and India (Kashmir). Apart from the commercial and historical aspects, several other characteristics make saffron an interesting biological system: the spice is derived from the stigmas of the flower (Figure 1A), which are harvested manually and subjected to desiccation. The main colors of saffron, crocetin and crocetin glycosides, and the main flavors, picrocrocin and safranal, are derived from the oxidative cleavage of the carotenoid, zeaxanthin [2,3] (Figure 1B). Saffron belongs to the Iridaceae (Liliales, Monocots) with poorly characterized genomes of relatively large size.

The characterization of the transcriptome of saffron stigmas is likely to shed light on several important biological phenomena: the molecular basis of flavor and color biogenesis in spices, the biology of the gynoecium, and the genomic organization of Iridaceae. For these reasons, we have undertaken the sequencing and bioinformatics characterization of Expressed Sequence Tags (ESTs) from saffron stigmas.

## Results and discussion

### Sequencing and assembly

An oriented cDNA library from mature saffron stigmas in lambda Uni-ZAP [2] was kindly provided by Prof. Bilal Camara, University of Strasbourg. The library was subjected to automated excision, and the cDNA inserts were subjected to PCR amplification and sequenced from the 5' end.

9,769 electropherograms were analyzed with the Phred program [4]. Low quality sequences were removed from the 5' and 3' ends, and the sequences were further processed to remove vector contaminations and to mask low complexity and/or repeat sub-sequences. This process reduced the original dataset to 6,603 high-quality sequences longer than 60 nucleotides. Only 6,202 EST fragments whose length is greater than or equal to 100 nucleotides were considered for the submission to the NCBI dbEST division. They are accessible under the accession numbers from EX142501 to EX148702.

**Figure 2:**
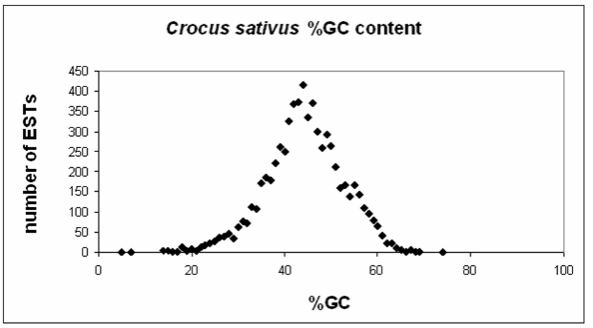
**GC content distribution**. The number of ESTs is plotted against their GC content. The average GC content is 44.3%.

The EST dataset was subjected to a clustering/assembling procedure [5], in order to group ESTs putatively derived from the same gene and to generate a tentative consensus sequence (TC) per putative transcript. The total number of clusters generated are 1,893. Each cluster should correspond to a unique gene, i.e. it represents a gene index. 1,376 clusters are made up of a single EST and are therefore classified as singletons. The remaining 517 clusters are made up of 5,324 ESTs, assembled into 534 TCs (Table 1). In 11 clusters, ESTs are assembled so that multiple TCs are defined (ranging from 2 to 6). Multiple TCs in a cluster have common regions of high similarity that may be due to possible alternative transcripts, to paralogy or to domain sharing. The GC content distribution in the dataset is reported in Figure 2. The average GC content is around 44%.

**Table 1:** Assembly statistics

Singleton ESTs	N. of sequences	1,376
	Avg. EST length (nt)	239
	Nucleotides masked	10.2%
		
ESTs in contigs	N. of sequences	5,324
	Avg. EST length (nt)	427
	Nucleotides masked	2.4%
		
Contigs	N. of contigs	534
	Avg. length (nt)	552

### The database and the web interface

The dataset was used to construct the *Saffron Genes *database [6]. The database architecture consists of a main MySQL relational database where all the data generated are deposited, and two satellite databases myGO and myKEGG. A user-friendly web interface is created using HTML and PHP scripts. A pre-defined query system supports data retrieval; HTML-tree graphical display is implemented to browse enzyme classes and metabolic pathways. Transcripts, which correspond to criteria defined by the user, can be mapped on-the-fly onto the KEGG metabolic maps, which are accessible as GIF images [7]. The electropherograms of the single ESTs can be downloaded to re-check sequence quality.

### Automated functional annotation

In order to assign a preliminary function to each transcript, the TCs and singletons were compared using BLASTX to the UniProtKB/Swiss-Prot database. Of 1,910 transcripts, 1,158 (60.6%) have no hits, while the remaining 752 (39.4%) have at least one significant match in the protein database. Within this latter set, 131 (6.9%) are described as hypothetical, unknown or expressed proteins thus not confirming an effective functional role of the transcript product.

**Figure 3:**
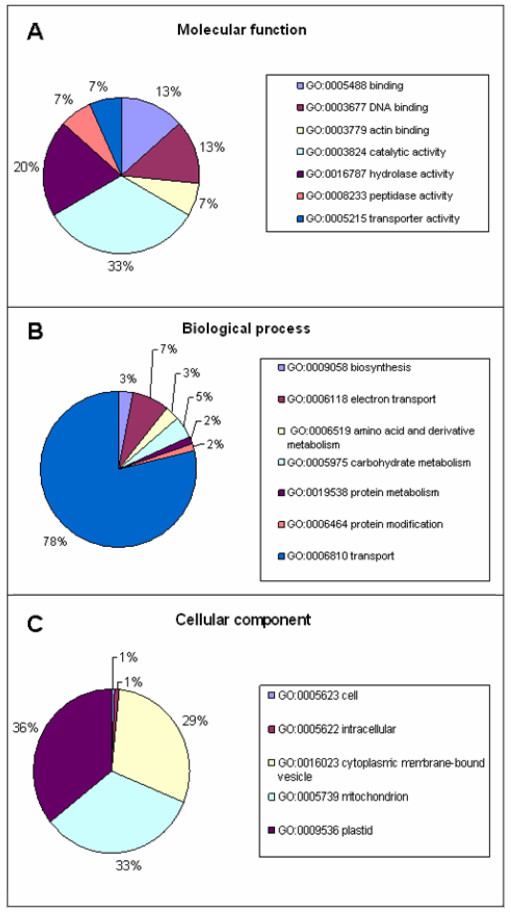
**Assignments of Plant Gene Ontology terms to the Crocus putative transcripts**. A. Molecular function B. Biological process C. Cellular component. For details, see Methods.

Gene Ontology terms were assigned automatically to those 157 transcripts matching a protein in the UniProtKB/Swiss-Prot database whose accession numbers are present into the satellite database myGO (see Methods). In many cases, multiple gene ontology terms could be assigned to the same transcript, resulting in 210 assignments to the molecular function, 944 to the biological process and finally 2,192 to the cellular component class. To give a broad overview of the ontology content, the entire set of the ontologies was mapped onto the plant GO Slims terms. In the molecular function ontology class, the most represented terms describe catalytic (33.3%) and hydrolase activity (20.0%) (Figure 3A). The remaining categories are less represented. Considering the biological process class, the vast majority of the GO assignments corresponds to the more general transport category (~78.8%) (Figure 3B). Finally, for the cellular component class the assignments were mainly given to the plastid (36%), mitochondrion (33%), and cytoplasmic membrane-bound vesicle (29%) components (Figure 3C). 64 transcripts are associated to 46 distinct enzymes as they are classified and described into the ENZYME repository [8]. 35 out of the 46 enzymes had mappings to 55 KEGG biochemical pathways [9]. As we know, some enzymes can occur in more than one pathway; on the other hand there are 8 enzymes which only act in a single pathway, that were classified as pathway-specific (data not shown).

### Genes expressed in Crocus stigmas

**Figure 4:**
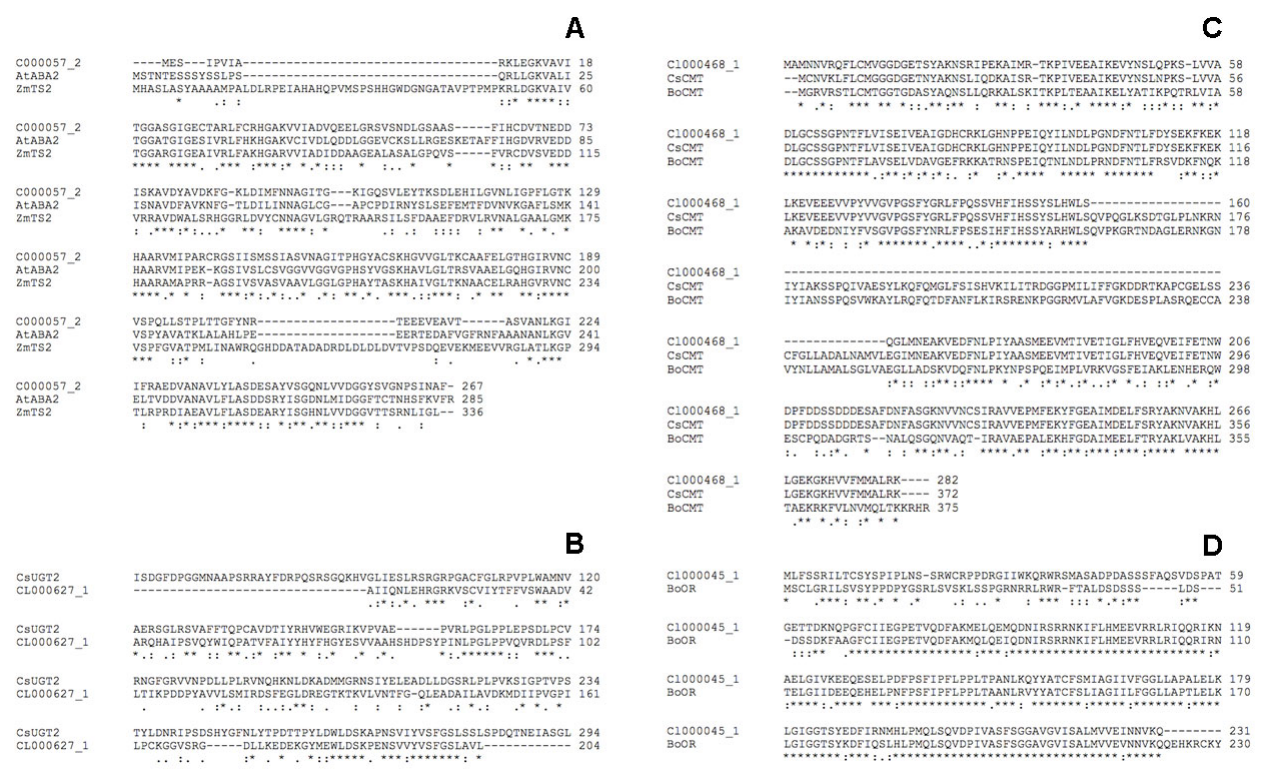
**ClustalW alignments of deduced protein sequences expressed in *Crocus *stigmas**. A. Cl000057:2, aligned with short chain alcohol dehydrogenases: Arabidopsis ABA2 (GenBank acc. NP_175644) and maize TS2 (GenBank acc. P50160). B. Cl000627:1, aligned with *Crocus *crocetin Glycosyltrasferase 2 (GenBank acc. P50160). C. Cl000468:1, aligned with *Bixa *and *Crocus *carboxyl methyltransferases (Genbank acc. CAD70190 and CAD70566) D. C1000045:1, aligned with cauliflower Or (GenBank acc. ABH07405).

EST abundance in a contig can be indicative of the mRNA relative abundance in the stigma tissue. We identified the TCs that are composed of ≥ 20 ESTs (Table 2). The most highly expressed TC, Cl000057:2 (547 ESTs), bears homology to short chain dehydrogenases (PF00106.12). This protein family comprises members involved in hormone biosynthesis, like the *ABA2 *gene of Arabidopsis which catalyzes the conversion of xanthoxin into ABA aldehyde [10], or in sexual organ identity, like the *TASSELSEED2 *(*TS2*) gene of maize (Figure 4). *TS2 *is expressed in pistil primordia cells of maize, where it activates a cell death process eliminating these cells from male reproductive organs [11]. Biochemical studies suggest that the TS2 protein is a hydroxysteroid dehydrogenase [12]. It will be interesting to determine the function and substrate specificity of the saffron Cl000057:2 product.

**Table 2:** Highly expressed TCs

**Contig**	**# ESTs**	**bp**	**BlastX annotation**	**e-value**
Cl000057:2	547	1242	Q7XL00_ORYSA -OJ000315_02.17 protein	0
Cl000837:2	122	1528	Q8VZY2_MUSAC -Cytochrome P450-1	0
Cl000799:2	114	711	-	-
Cl001953:2	109	755	O80821_ARATH -Hypothetical protein At2g41470	1,00E-16
Cl001114:3	104	770	HSP13_ARATH -18.2 kDa class I heat shock protein (HSP 18.2)	1,00E-32
Cl000299:1	104	570	Q9XHD5_IPOBA -B12D protein	2,00E-32
Cl000870:1	94	592	Q6ZX06_ORYSA -Lipid transfer protein	3,00E-26
Cl001582:1	61	600	-	-
Cl000209:1	61	1071	Q5G1M8_9POTV -Polyprotein (Fragment)	0
Cl001173:1	56	785	Q6H452_ORYSA -Putative monoglyceride lipase	0
Cl000220:1	55	831	Q94HY3_ORYSA -Putative gamma-lyase	0
Cl000348:1	54	955	Q9AVB7_9LILI -LhMyb protein	0
Cl001319:1	47	460	Q8RVT5_PANGI -Acyl-CoA-binding protein	1,00E-35
Cl001051:1	45	665	Q8H293_ANACO -Cytochrome b5	0
Cl000246:1	45	537	-	-
Cl000336:1	44	685	GPAT6_ARATH -Glycerol-3-phosphate acyltransferase 6 (EC 2.3.1.15)	0
Cl000468:2	42	1021	Q70SZ8_9ASPA -Carboxyl methyltransferase	0
Cl000482:1	38	730	Q84P95_ORYSA -Disulfide isomerase	0
Cl000982:1	38	230	-	-
Cl001040:1	37	734	Q8GZR6_LYCES -GcpE	0
Cl001329:1	36	384	Q4LEZ4_ASPOF -MADS-box transcription factor	1,00E-29
Cl001815:1	34	992	BGAL_ASPOF -Beta-galactosidase precursor (EC 3.2.1.23) (Lactase)	0
Cl000113:1	33	634	Q6VAB3_STERE -UDP-glycosyltransferase 85A8	9,00E-16
Cl000687:1	33	782	Q9XGS6_PRUDU -Cytosolic class II low molecular weight heat shock protein	0
Cl000887:1	33	802	Q9FVZ7_ORYSA -Putative steroid membrane binding protein	0
Cl001463:1	32	605	Q9FE65_ARATH -60S ribosomal protein L34, putative	0
Cl000932:1	32	974	Q652L6_ORYSA -Putative monodehydroascorbate reductase	0
Cl001812:1	30	554	Q42338_ARATH -B12D-like protein	5,00E-32
Cl001134:1	29	569	Q8W453_ARATH -Hypothetical protein (DIR1 protein) (At5g48485)	7,00E-14
Cl001906:1	28	602	Q4TES1_TETNG -Chromosome undetermined SCAF5157	9,00E-07
Cl001988:1	25	1446	Q8VX49_WHEAT -Cytochrome P450 reductase (EC 1.6.2.4)	0
Cl001107:1	24	783	Q9SGA5_ARATH -F1C9.14 protein (At3g02070)	0
Cl001447:1	24	453	Q5VS45_ORYSA -Hypothetical protein P0425F02.23	1,00E-12
Cl000515:1	24	506	Q6ZCF3_ORYSA -Putative copper chaperone	8,00E-15
Cl000762:1	24	247	-	-
Cl001114:2	23	748	HSP13_ARATH -18.2 kDa class I heat shock protein (HSP 18.2)	1,00E-32
Cl001894:1	23	312	-	-
Cl000057:1	23	740	TRXH1_ARATH -Thioredoxin H-type 1 (TRX-H-1)	1,00E-36
Cl001263:1	22	667	Q9XH76_ARATH -Zinc finger protein-like (PMZ)	0
Cl001010:1	21	1066	Q8H2A7_ANACO -PFE18 protein (Fragment)	0
Cl000300:1	21	506	Q93WW3_NARPS -Metallothionein-like protein type 2	6,00E-12
Cl000057:3	21	183	-	-
Cl000885:2	21	753	Q41067_PINSY -Polyubiquitin	0
Cl001397:1	20	798	Q9LSQ5_ARATH -1,4-benzoquinone reductase-like;	0
Cl001774:1	20	457	Q9SN96_ARATH -Hypothetical protein F18L15.150	7,00E-19
Cl000185:1	20	397	Q84LB7_MALDO -Cysteine protease inhibitor cystatin (Fragment)	2,00E-12
Cl001935:1	20	673	SRP19_ARATH -Signal recognition particle 19 kDa protein (SRP19)	4,00E-38
Cl000333:1	20	418	Q7F6G0_ORYSA -Putative metallothionein-like protein	6,00E-20
Cl000594:1	20	1145	SUS1_TULGE -Sucrose synthase 1 (EC 2.4.1.13)	0

A large number of Cytochrome P450 sequences are expressed in saffron stigmas, some of which at very high levels (Tables 2 and 3). Also, lipid metabolism seems to be very active, judging from the TCs encoding proteins involved in this process (Table 3).

**Table 3:** TCs grouped by putative function

**Contig**	**# ESTs**	**bp**	**BlastX annotation**	**e-value**
**Cyt. P450**				

cr.saCl000837:2	122	1528	Q8VZY2_MUSAC – Cytochrome P450-1	0
cr.saCl001988:1	25	1446	Q8VX49_WHEAT – Cytochrome P450 reductase (EC 1.6.2.4)	0
cr.saCl000837:3	17	674	Q8L5Q2_CICAR – Putative cytochrome P450 monooxygenase	2e-27
cr.saCl000414:1	5	752	Q9AVM1_ASPOF – Cytochrome P450	0
cr.saCl000150:1	3	406	Q9ATU9_LOLRI – Putative cytochrome P450	4e-17
cr.saCl000166:1	3	710	Q6EP96_ORYSA – Putative cytochrome P450	9e-16
cr.saCl001887:1	2	248	Q6H516_ORYSA – Putative cytochrome P450	0.0004
cr.saCl000837:1	2	600	Q8VZY2_MUSAC – Cytochrome P450-1	3e-16
cr13_O11	1	360	Q8S7S6_ORYSA – Cytochrome P450-like protein	7e-35
cr21_F05	1	448	Q8S7S6_ORYSA – Cytochrome P450-like protein	1.00053e-42
cr28_M16	1	533	Q6Z0U4_ORYSA – Putative cytochrome P450 reductase	0
cr34_J15	1	509	Q8S7S6_ORYSA – Cytochrome P450-like protein	0

**Lipid metabolism**				

cr.saCl000870:1	94	592	Q6ZX06_ORYSA – Lipid transfer protein	3e-26
cr.saCl001173:1	56	785	Q6H452_ORYSA -Putative monoglyceride lipase	0
cr.saCl000787:1	10	743	Q94GF2_ORYSA – Putative phospholipase	0
cr.saCl001992:1	5	637	Q52RN7_LEOAR – Non-specific lipid transfer protein-like	2e-28
cr.saCl001009:1	5	667	O04439_ALLPO – 3-ketoacyl carrier protein synthase III	0
cr.saCl001749:1	5	635	Q9NCL8_DICDI – Phosphatidylinositol transfer protein 1	5e-30
cr.saCl000344:1	5	704	O49902_NICRU – 1-phosphatidylinositol-4,5-bisphosphate phosphodiesterase	0
cr.saCl000816:1	2	677	Q6K7T9_ORYSA – Peroxisomal fatty acid beta-oxidation multif. protein	0
cr.saCl000294:1	2	707	Q84Z91_ORYSA – Oxysterol-binding protein-like	0
cr.saCl000741:1	2	734	STAD_ORYSA – Acyl-(acyl-carrier-protein) desaturase, chloroplast precursor	0
cr13_F23	1	350	Q8S459_LYCES – Putative sphingolipid delta 4 desaturase DES-1	0
cr15_P04	1	306	GPX4_MESCR – Probable phospholipid hydroperoxide glutathione peroxidase	5e-16
cr27_P08	1	74	Q5N7U2_ORYSA – Phospholipid/glycerol acyltransferase-like protein	4e-06
cr35_M17	1	437	GPX4_MESCR – Probable phospholipid hydroperoxide glutathione peroxidase	1e-24

**Carotenoid metabolism**				

cr.saCl000944:1	11	645	Q8VXP2_9ASPA – Beta-carotene hydroxylase	4e-17
cr.saCl001432:1	2	602	Q9FZ04_CAPAN – Plastid terminal oxidase	0
cr.saCl001532:1	7	420	GT_CITUN – Limonoid UDP-glucosyltransferase	2e-06
cr.saCl001032:1	2	426	5CD69_9MYRT – Monoterpene glucosyltransferase	2e-08
cr.saCl000627:1	2	611	69UF5_ORYSA – Putative anthocyanin 5-O-glucosyltransferase	0
cr.saCl000468:2	42	1021	Q70SZ8_9ASPA – Carboxyl methyltransferase	0
cr.saCl000468:1	6	767	70SZ8_9ASPA – Carboxyl methyltransferase	0
cr9_J02	1	69	Q9FEC9_LYCES – Plastid quinol oxidase (Plastid terminal oxidase)	1e-05
cr36_B21	1	706	PAP2_ORYSA – Probable plastid-lipid associated protein 2, chloroplast precursor	0
cr.saCl000045	14	746	Q9FKF4_ARATH – Hypothetical protein At5g61670	0

**Transcription factors**				

cr.saCl000348:1	54	955	Q9AVB7_9LILI – LhMyb protein	0
cr.saCl001329:1	36	384	Q4LEZ4_ASPOF – MADS-box transcription factor	1e-29
cr.saCl000348:2	6	669	Q70RD2_GERHY – MYB8 protein	0
cr.saCl000712:1	6	714	Q6Z8N9_ORYSA – Putative AT-hook DNA-binding protein	0
cr.saCl000359:1	5	593	O82115_ORYSA – Zinc finger protein	5e-19
cr.saCl000502:1	3	565	ULT1_ARATH – Protein ULTRAPETALA1	4e-37
cr.saCl000652:1	2	537	Q6ZG02_ORYSA – Putative DNA-binding protein WRKY2	0
cr17_J15	1	567	Q6Q6W8_9ASPA – Agamous MADS-box transcription factor 1a	0
cr26_B12	1	653	Q8LAP4_ARATH – Similar to MYB-related DNA-binding protein	2e-23
cr6_B13	1	312	Q9M7F3_MAIZE – LIM transcription factor homolog	0

Several TCs encode putative carotenoid metabolism enzymes (Table 3): Cl000944:1 encodes non-heme -β-carotene-hydroxylase, which is highly expressed in saffron stigmas [13]. Cl000627:1 encodes a putative glucosyltransferase, very similar to UGTCs2, which is able to glycosylate crocetin in vitro [3] (Figure 4). Cl001532:1 and Cl001032:1 also, encode putative isoprenoid GTases, one of which could represent the still missing enzyme responsible for the glycosylation of picrocrocin (Figure 1). Cl001432:1 encodes a protein similar to plastid terminal oxidase, involved in phytoene desaturation [14], while EST cr36_B21 encodes a protein similar to fibrillin, which is a carotenoid-binding protein in pepper chromoplasts [15]. Cl000468 encodes a carboxyl methyltransferase very similar to the one catalyzing the synthesis of bixin [16] (Figure 4). This TC seems to encode a "short" form of the annatto and crocus methyltransferases from GenBank, possibly derived from alternative splicing (Figure 4). Although a methyltransferase reaction has not been described in saffron stigmas, the biosynthesis of bixin and that of crocin share some features in common, since both pigments are derived from the oxidative cleavage of a carotenoid [17]. Finally, Cl000045:1 encodes a protein highly similar to the cauliflower *Or *gene product, a plastid-associated protein with a cysteine-rich DnaJ domain. A dominant *Or *mutation induces β-carotene accumulation in cauliflower inflorescences, suggesting that *Or *is somehow involved in the control of chromoplast differentiation [18,19].

Several TCs encode putative transcription factors (Table 3). The most abundantly expressed, Cl000348:1, encodes a Myb-like protein with high similarity to LhMyb (from *Lilium*, GenBank accession BAB40790) Myb8 (from *Gerbera *[20] – also showing similarity to Cl000348:2) and Myb305 (From *Antirrhinium *[21]). All three factors are highly expressed in flowers. Also highly expressed is Cl001329:1, encoding a putative MADS box transcription factor. This protein shows high similarity to AODEF, a B-functional transcription factor from *Asparagus *expressed in stamens and inner tepals [22] and to LMADS1, a lily protein whose ectopic expression in dominant negative form causes an *ap3*-like phenotype in Arabidopsis [23].

Finally, several TCs – Cl000209:1 (61 ESTs) Cl000582:1 (18 ESTs) Cl001827:1 (5 ESTs) and Cl000731(2 ESTs) – show similarity to potyviral sequences, indicating that the sequenced library likely derives from virus-infected tissue. Potyviruses like Iris Mild Mosaic Virus are known to infect *Crocus *[24]. The sequences of these TCs will prove useful for diagnostic and phytosanitary purposes.

## Conclusion

The *Saffron Genes *database [6] has been designed to manage and to explore the EST collection from saffron stigmas, providing a reference for the expression pattern analysis in this tissue as well as a primary view of the genomic properties of this species, representative of Iridaceae. The complete set of raw EST sequences, as well as of their electopherograms, are maintained in the database allowing users investigate on library qualities and on single EST structural features (vector contamination, repeat regions). Annotation is provided for single ESTs as well as for their assemblies (tentative consensus), to evaluate the consistency of the automated functional assignments. The putative transcripts determined to be associated to enzymes are organized into classes and can be viewed also in terms of enzyme assignments to metabolic pathways. This represents a straightforward way to investigate the properties of the stigma transriptome. As discussed above, this transcriptome contains a series of interesting sequences, whose function can now be tested using in vivo or in vitro approaches.

## Methods

### EST sequencing

An oriented cDNA library from mature saffron stigmas in lambda Uni-ZAP [2] was kindly provided by Prof. Bilal Camara, University of Strasbourg. The pBluescript phagemids contained in the phages were subjected to in vivo excision using the ExAssist helper phage and the SOLR strain according to the manufacturer's protocols (Stratagene Uni-ZAP manual) and plated on LB Agar plates with Ampicillin, IPTG and X-GAL. White colonies were picked by hand and grown overnight in LB 384-well plates in LB+Ampicillin. Approx. 1 uL of each saturated culture was inoculated using a 384-pin tool (VP Scientific) in a 20-uL PCR reaction containing 50 ng each of primers T3 and T7 (Stratagene) and 0.5 U Taq Polymerase (GE Healthcare). The reactions, in 384-well format, were amplified using the following PCR cycle: denaturation step (94°C 2'), followed by 35 cycles of denaturation (94°C 45") annealing (50°C 45") and elongation (72°C 2'), followed by an elongation step (72°C 10'). Approx. 1/4 of the PCR reactions were checked by loading 2 mL on a 1% agarose gel, and only plates containing > 80% amplified, robust single bands were processed further. The PCR reactions were purified by gel filtration on 384-well deep-well PVDF plates (Corning cat. 3531). Each well was filled with 250 mL of resin (3.5% Sephadex G-100, GE Healthcare) and the resin was packed by centrifugation at 3.000 × g for 5'; after addition of 150 mL of resin, the plate was re-centrifuged as above; 10 mL of the PCR template were loaded in each well and the purified reaction was collected by centrifugation as above.

3 mL of the PCR template were used for sequencing with the T3 primer in a final volume of 10 mL in 384-well format. The BigDye Terminator kit v 3.1 (Applied Biosystems) was used according to the manufacturer's instructions at a dilution of 1:16. The dye terminators were removed by gel filtration on 384-well deep-well PVDF plates (see above) using 6.5% Sephadex G-50 fine (GE Healthcare). The reactions were loaded on an ABI 3730 sequencer with 50-cm capillaries.

### EST processing and contig assembly

The electropherograms were analyzed using the pipeline ParPEST developed at the University of Naples [5]. Sequence base calls were performed using Phred [4] with a quality cutoff of 0.05. Vector contaminations were identified using RepeatMasker [25] and NCBI's UniVec as filtering database. RepeatMasker and RepBase [26] are used for filtering and masking low complexity sub-sequences and interspersed repeats. EST clustering was made using PaCE [27] with default parameters. All the ESTs in a cluster are assembled into contigs using CAP3 [28] with an overlapping window of 60 nucleotides and a minimum score of 85.

### Functional annotation

Raw EST data and contigs are compared using BLASTX against the UniProtKB/Swiss-Prot database [29]. The BLAST search is filtered setting an e-Value less equal than 0.001. The association between the transcripts and the Gene Ontology terms occurs when the accession number of the protein subject is reported in the myGO database. All the GO terms related to each best BLAST hit were converted to the plant GO Slim terms using the map2slim.pl script, distributed as part of the go-perl package (version 0.04). The plant GO Slim file was downloaded from ... the Gene Ontology webpage [30]. The association between the transcripts and the Enzyme Commission (EC) numbers occurs if the EC is present in the description lines of each best BLAST hit. Transcripts, which are associated to EC numbers, are also linked to myKEGG and can be mapped onto the metabolic pathways.

### Multiple alignment generation

ClustalW sequence alignment [31] was performed using the EBI web interface [32].

## Competing interests

The authors declare that there are no competing interests.

## Authors' contributions

GG planned and supervised the entire work. DP performed the sequencing. MLC planned and supervised the bioinformatics work which was implemented by NDA. GG and MLC wrote the paper. All authors read and approved the final manuscript.
